# Machine Learning for Building Immune Genetic Model in Hepatocellular Carcinoma Patients

**DOI:** 10.1155/2021/6676537

**Published:** 2021-03-17

**Authors:** Jun Liu, Zheng Chen, Wenli Li

**Affiliations:** ^1^Reproductive Medicine Center, Yue Bei People's Hospital, Shantou University Medical College, Shaoguan, Guangdong, China; ^2^Medical Research Center, Yue Bei People's Hospital, Shantou University Medical College, Shaoguan 512025, China; ^3^Liver Cancer Institute, Zhongshan Hospital, Fudan University, Key Laboratory of Carcinogenesis and Cancer Invasion, Ministry of Education, Shanghai, China

## Abstract

**Background:**

Hepatocellular carcinoma (HCC) is the leading liver cancer with special immune microenvironment, which played vital roles in tumor relapse and poor drug responses. In this study, we aimed to explore the prognostic immune signatures in HCC and tried to construct an immune-risk model for patient evaluation.

**Methods:**

RNA sequencing profiles of HCC patients were collected from the cancer genome Atlas (TCGA), international cancer genome consortium (ICGC), and gene expression omnibus (GEO) databases (GSE14520). Differentially expressed immune genes, derived from ImmPort database and MSigDB signaling pathway lists, between tumor and normal tissues were analyzed with Limma package in *R* environment. Univariate Cox regression was performed to find survival-related immune genes in TCGA dataset, and in further random forest algorithm analysis, significantly changed immune genes were used to generate a multivariate Cox model to calculate the corresponding immune-risk score. The model was examined in the other two datasets with recipient operation curve (ROC) and survival analysis. Risk effects of immune-risk score and clinical characteristics of patients were individually evaluated, and significant factors were then used to generate a nomogram.

**Results:**

There were 52 downregulated and 259 upregulated immune genes between tumor and relatively normal tissues, and the final immune-risk model (based on SPP1, BRD8, NDRG1, KITLG, HSPA4, TRAF3, ITGAV and MAP4K2) can better differentiate patients into high and low immune-risk subpopulations, in which high score patients showed worse outcomes after resection (*p* < 0.05). The differentially enriched pathways between the two groups were mainly about cell proliferation and cytokine production, and calculated immune-risk score was also highly correlated with immune infiltration levels. The nomogram, constructed with immune-risk score and tumor stages, showed high accuracy and clinical benefits in prediction of 1-, 3- and 5-year overall survival, which is useful in clinical practice.

**Conclusion:**

The immune-risk model, based on expression of SPP1, BRD8, NDRG1, KITLG, HSPA4, TRAF3, ITGAV, and MAP4K2, can better differentiate patients into high and low immune-risk groups. Combined nomogram, using immune-risk score and tumor stages, could make accurate prediction of 1-, 3- and 5-year survival in HCC patients.

## 1. Introduction

Hepatocellular carcinoma (HCC) is one of the most malignant tumors around the world, causing the second highest mortal rate with poor responses to therapies [1, 2]. The immune microenvironment of tumors has been testified to play vital roles in tumor progression and relapse, which incurred development of immune therapies, such as application of checkpoint inhibitors and Car-T transfusion [3]. Though these drugs, approved by the federal government in several countries, demonstrated efficacy in tumor regression and prolonged overall survival, overall response was not satisfying in patients, which may be related to tumor mutation burden and immune infiltration levels [4]. Understanding immune microenvironment within HCC can better predict patients' survival, which can also be used to guide drug usage or treating strategies.

There have been some studies investigating prognostic signatures of HCC; however, high-precision prognostic models based on immune-related genes were few in HCC [5, 6]. In this study, we managed to construct an immune-risk model to differentiate HCC patients into high- and low-risk subgroups for survival prediction. High score patients had worse prognosis after resection, and the significantly changed immune genes between HCC tissues and normal tissues were highly enriched in pathways of cell growth and tyrosine kinase inhibitor resistance. Combining immune-risk score and tumor stage, we constructed a nomogram with high precision, which can help therapists make comprehensive clinical evaluation of patients in practice.

## 2. Materials and Methods

### 2.1. Data Description and Derivation of Immune Gene List

Expression data included in the analysis were from TCGA database (https://www.cancer.gov/about-nci/organization/ccg/research/structural-genomics/tcga), ICGA database (https://icgc.org), and GEO database (https://www.ncbi.nlm.nih.gov/geo) [7, 8]. Clinical information of all the related patients (TCGA: 370 cases, GSE14520: 209 cases, ICGC: 232 cases) was shown in Table 1. Immune gene list analyzed in this study was procured from ImmPort database (https://www.immport.org) and MSigDB pathway signature list (IMMUNE_RESPONSE and IMMUNE_SYSTEM_PROCESS). The gene list of transcription factors was from CISTROME Project (http://cistrome.org/), which is an open web tool for tumor sequencing data analysis.

### 2.2. Analysis of Differentially Expressed Immune Genes

RNA expression levels of different genes from patients in TCGA dataset were analyzed between the hepatocellular carcinoma tissues and the para-tumor tissues, using Limma package in *R* environment [9]. The genes expressing more than 1-fold change with adjusted *p* value under 0.05 were considered significant after normalization and background correction. The package of Heatmap was used to create the heatmap of significantly up- and downregulated genes.

### 2.3. Gene Ontology and Gene Enrichment

To better understand the functional roles of the significantly changed genes, the package of clusterProfiler was used to demonstrate the gene ontology and enriched pathways, including the cellular compartment, biological process, molecular function, and KEGG pathways (Kyoto Encyclopedia of Genes and Genomes, https://www.kegg.jp) [10]. The further gene set enrichment analysis (GSEA) between high and low immune-score patients was performed with GSEA 4.0.1 software in order to locate the related gene sets [11, 12].

### 2.4. Construction of Immune Prognostic Models and Nomogram

The RNA sequencing data in TCGA LIHC dataset were used to find the differentially expressed immune genes, and through univariate Cox regression, survival-related immune genes (overall survival and progression free survival) (*p* < 0.05) were selected for risk evaluation. The overlapped gene set (68 signatures) was chosen to find the most relevant prognostic genes, and random forest algorithm was used to compress the gene list. The random forest algorithm was performed to determine the important of immune-related genes, and the relative importance >0.2 was identified as the final signature. The finally yielded 8 immune genes (SPP1, BRD8, NDRG1, KITLG, HSPA4, TRAF3, ITGAV, and MAP4K2) were also evaluated for their variable relative importance and were further put into multivariate Cox analysis to generate the immune-risk model. These analyses were performed with *R* package of randomForestSRC and algorithm of randomSurvivalForest. In construction of the model, median risk score (0.961) was deployed to assign patients into high and low immune-risk groups, and, correspondingly, prognostic value of the model was examined in the training set of TCGA and the testing sets of ICGC and GSE14520 (immune-risk score = 0.115^*∗*^expSPP1 + 0.263^*∗*^ expBRD8 + 0.125^*∗*^ expNDRG1 + 0.233^*∗*^ KITLG + 0.195^*∗*^ HSPA4 + 0.319^*∗*^ TRAF3 − 0.186^*∗*^expITGAV + 0.333expMAP4K2).

Immune-risk score and clinical characteristics of patients, such as age, gender, tumor grades, and stages, were then put into Cox model to evaluate corresponding risk effect. Immune-risk score and tumor stages were significantly correlated with survival of patients before or after adjustment, which were then used to create a nomogram for prognosis prediction in 1-, 3-, and 5-year follow-up. C-index of the nomogram was calculated with bootstrap of 1000 resamples, ranging from 0.5 to 1.0. Precision of the model was examined by calibration graphs, in which the alignment of both lines was a fact of good performance, reflecting the actual probability. Recipient operation curves (ROCs) were used to demonstrate its specificity and sensitivity in comparison with other factors. Decision curve analysis (DCA) was also deployed to evaluate the potential clinical benefits of the model. ROC and DCA were performed with *R* packages of rms, survcomp, and survivalROC. Kaplan–Meier (K-M) method was used to estimate the accumulative incidence of survival between different subpopulations in each dataset. The survival curves were drawn through *R* package of survival and compared with log-rank test.

### 2.5. Analysis of the Infiltrated Immune Cells

The infiltration of six common immune cells was estimated through online database of TIMER (https://cistrome.shinyapps.io/timer/), which is an open tool with data access to 32 types of cancers in TCGA database [13, 14]. Infiltration levels of B cells, CD4+ T cells, CD8+ T cells, neutrophils, dendritic cells, and macrophages were calculated correspondingly, and the correlation between immune-risk score and infiltrated cell types was examined by Pearson's correlation test. Evaluation of immune status's difference between high and low immune-risk patients was performed with single sample gene set enrichment analysis (ssGSEA), using gene sets in MSigDB pathway lists. Expression of well-known immune checkpoints was also compared between groups. *p* value under 0.05 was considered significant.

## 3. Results

### 3.1. Differentially Expressed Immune Genes between HCC and Para-Tumor Tissues

**Figure 1:**
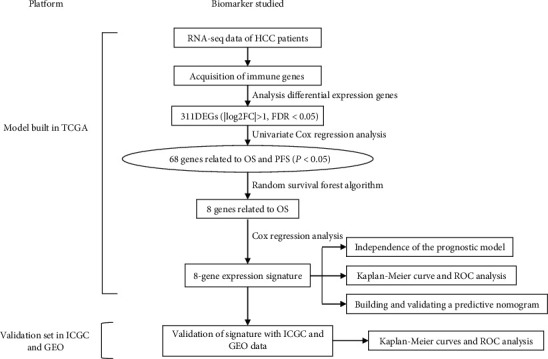
Workflow of the analysis.

**Figure 2:**
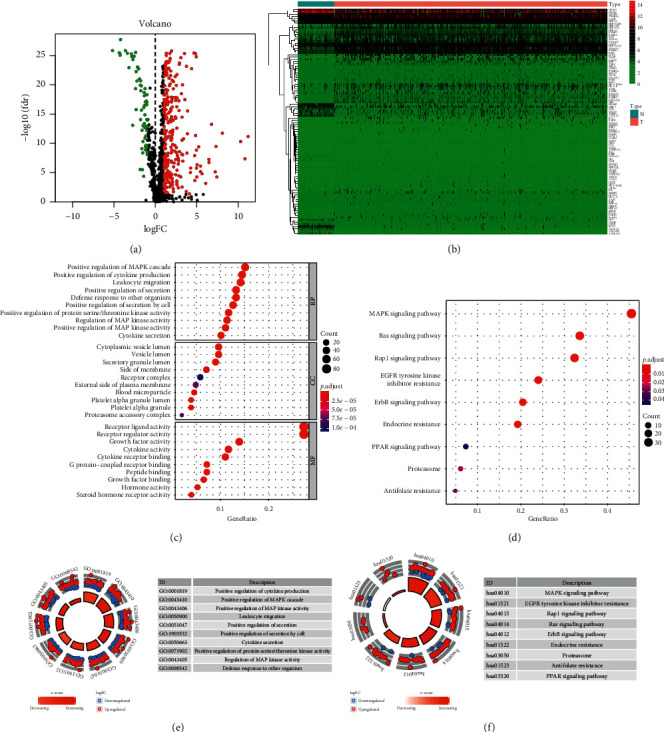
Differentially expressed immune genes (DEIGs) and gene ontology. (a). Volcano graph of DEIGs between HCC and para-tumor tissues. (b) Heatmap of DEIGs. (c) Gene ontology of DEIGs. (d) Enriched pathways for DEIGs through KEGG website. (e) Top ranked gene ontology for DEIGs. (f) Top ranked KEGG pathways for DEIGs.

Work flow of this study was shown in Figure 1. We found 311 differentially expressed immune genes between HCC and para-tumor tissues (log2-transformed fold change> 1, *p* < 0.05). In the list of significantly changed genes, 259 genes were upregulated and 52 genes were downregulated (Figures 2(a) and 2(b)). Gene ontology of those significantly changed genes was mainly involved in signal transduction and cytokine regulation (Figures 2(c) and 2(e)). The highly enriched pathways were MAPK signaling pathway, Rap1 signaling pathway, Ras signaling pathway, EGFR tyrosine kinase inhibitor resistance, ErbB signaling pathway, and so on, which were well-known signalings for tumor growths and metastasis, indicating the protumor immune microenvironment (Figures 2(d) and 2(f)). We also analyzed the differentially expressed transcription factors (TFs) between normal and tumor tissues in HCC patients (Supplemental Figures 1(a) and 1(b)). It turned out most TFs were upregulated in tumor tissues (108 upregulated, 9 downregulated).

Constructed immune-risk model with eight signatures can better stratify patients into subpopulations.

**Figure 3:**
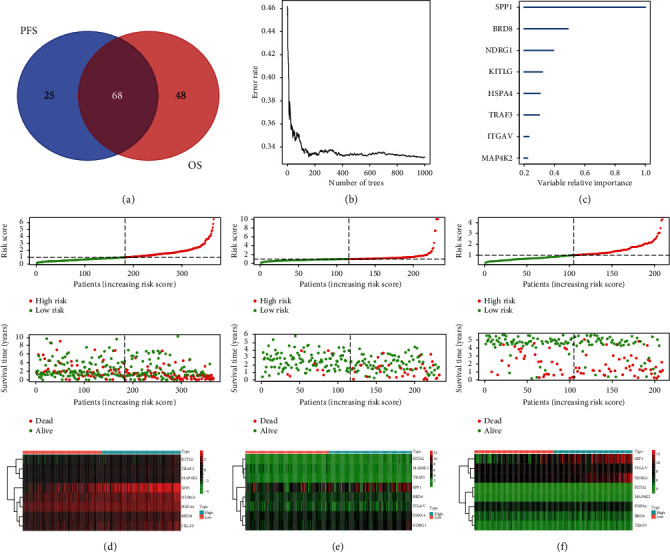
Construction of immune-risk model with DEIGs and further assignment of patients. (a) Venn diagram for survival-related (OS and PFS) DEIGs in TCGA dataset. (b) Random forest algorithm was used to downsize the survival-related DEIGs. (c) The relative importance for eight immune signatures generated by random forest algorithm. (d–f) The division of patients with median risk score calculated by 8-gene immune-risk model and the corresponding data spread in training dataset from TCGA and testing datasets from ICGC and GSE14520. The expression difference of the eight signatures between high and low immune-risk groups was shown by heatmap.

We performed univariate Cox analysis to locate survival-related genes in the training set from TCGA database. In association with overall survival (OS), we located 93 signatures, while in association with progression free survival (PFS), we located 116 signatures. The overlapped 68 signatures were used to build immune-risk model (Figure 3(a)). The regulating network between differentially expressed TFs and survival-related immune genes was also examined (Supplemental Figure 1(c)).

**Figure 4:**
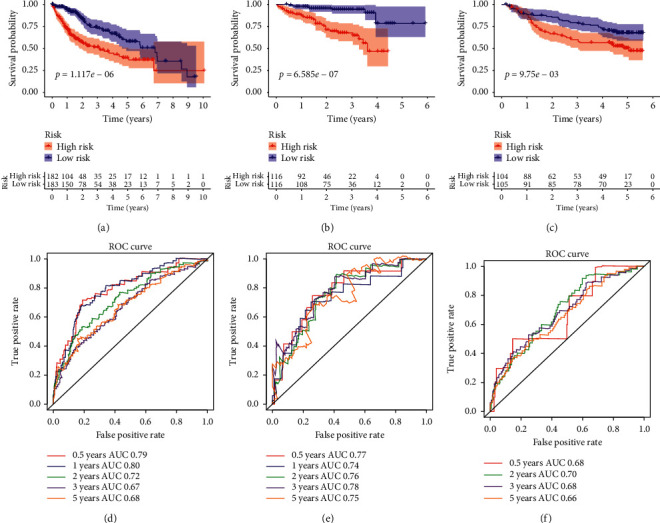
The overall survival difference between high and low immune-risk score patients in datasets of TCGA, ICGC, and GSE14520, and their respective predicting values for 0.5, 1-, 2-, 3-, and 5-year survival. (a–c) Overall survival difference between high and low immune-risk patients in TCGA, ICGC, and GSE14520 datasets. (d–f) Recipient operation curve (ROC) for 0.5-, 1-, 2-, 3-, and 5-year survival prediction of immune-risk model in patients from TCGA, ICGC, and GSE14520 datasets.

Random forest algorithm was used to additionally downsize the candidate signatures, and after iteration, signatures of SPP1, BRD8, NDRG1, KITLG, HSPA4, TRAF3, ITGAV, and MAP4K2 were used to construct immune-risk model (Figures 3(b) and 3(c)). Through multivariate Cox regression, we used the immune-risk model to calculate the immune-risk score of HCC patients, and using the mean immune-risk score, we dichotomized patients into high- and low-risk subpopulations (Figures 3(d), 3(e), and 3(f)). The difference of OS between high and low immune-risk score subgroups was consistent among all three datasets (TCGA, ICGC, and GSE14520), with high-risk patients suffering from poor outcomes in the follow-up (*p* < 0.05) (Figures 4(a)–4(c)). The area under curves (AUCs) for the immune-risk model in prediction of 0.5, 1-, 2-, 3-, and 5-year OS were 0.79, 0.80, 0.72, 0.67, and 0.68 in training set of TCGA dataset (Figure 4(d)). In testing dataset of ICGC, the corresponding AUC values were 0.77, 0.74, 0.76, 0.78, and 0.75, while, in GSE14520, AUCs for 0.5-, 2-, 3-, and 5-year OS were 0.68, 0.70, 0.68, and 0.66 (Figures 4(e) and 4(f)). In analysis of PFS, high immune-risk patients in TCGA dataset also suffered from worse outcomes, and the respective 0.5-, 1-, 2-, 3-, and 5-year AUCs were 0.64, 0.66, 0.63, 0.64, and 0.62 (Supplemental Figures 2(a) and 2(b)).

Independent risk effect for the immune-risk model and corresponding expression levels in patients' subgroups.

**Figure 5:**
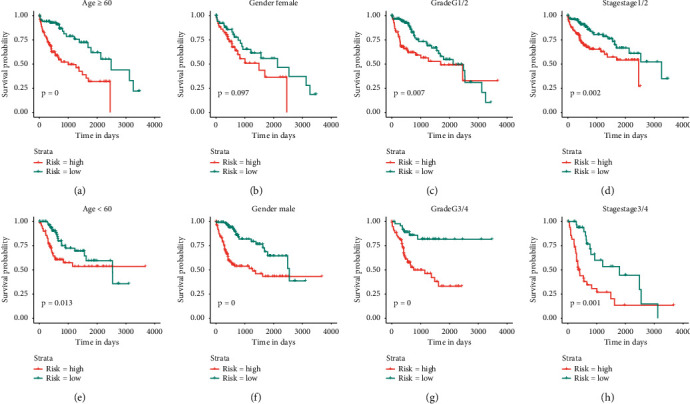
Overall survival (OS) difference between high and low immune-risk score patients in subpopulations with different clinical characteristics. (a-b) OS difference between high and low immune-risk patients in subgroups with age over or below 60 from TCGA dataset. (c-d) OS difference between high and low immune-risk patients in subgroups with different gender (female and male) from TCGA dataset. (e-f) OS difference between high and low immune-risk subgroups with tumor grades of grade 1/2 or grade 3/4 from TCGA dataset. (g-h) OS difference between high and low immune-risk patients in subgroups with tumor stage 1/2 or stage 3/4 from TCGA dataset.

Then we tried to evaluate the risk effect of immune-risk score and other clinical characteristics in relation to OS in TCGA dataset. After assignment of patients according to age (≥60 or <60), gender, and tumor grades (G1/2 or G3/4) and tumor stages (stage1/2 or stage3/4), we found high immune-risk patients shared worse outcomes consistently (Figures 5(a)–5(h)). Also, the respective results in relation to PFS were similar for HCC patients in TCGA dataset, in which high immune-risk patients shared shorter PFS time (Supplemental Figure 2(c)). All those indicated immune-risk score was an independent risk factor for HCC patients.

**Figure 6:**
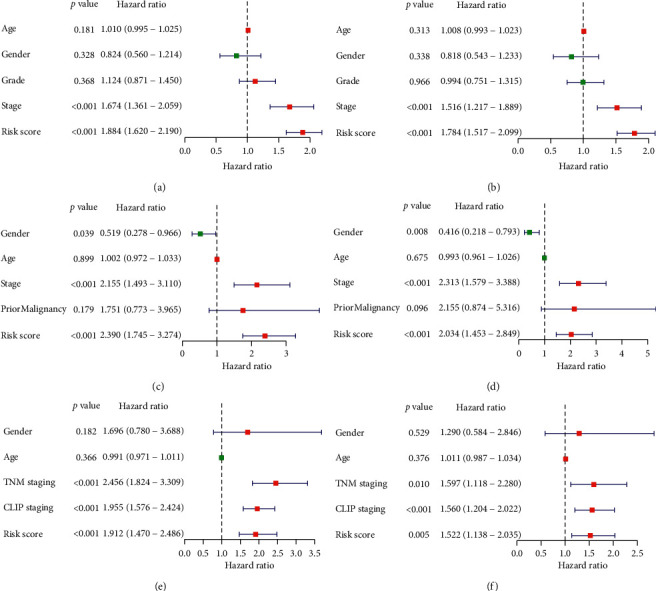
Univariate and multivariate analysis of overall survival-related risk factors in datasets of TCGA, ICGC, and GSE14520 (a, c, e). Univariate Cox analysis of the survival-related factors in TCGA, ICGC, and GSE14520 datasets (b, d, f). Multivariate Cox analysis of the survival-related factors in TCGA, ICGC and GSE14520 dataset.

We further examined clinical characteristics in single variate and multivariate Cox analysis in combination with immune-risk score. It showed in TCGA, ICGC, and GSE14520 datasets, tumor stages and immune-risk score were both significant risk factors to OS even after adjustment in multivariate model (Figures 6(a)–6(f)).

Enriched pathways in high immune-risk score patients demonstrated unique biological behaviors of HCC cells.

**Figure 7:**
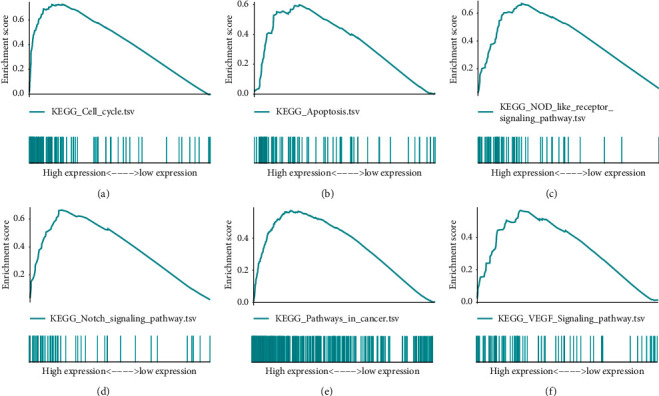
Highly enriched pathways in high immune-risk score patients through gene set enrichment analysis (GSEA). (a) Enriched score for signatures in the pathway of cell cycle. (b) Enriched score for signatures in the pathway of apoptosis. (c) Enriched score for signatures in the pathway of NOD like receptor signaling. (d) Enriched score for signatures in the pathway of Notch signaling. (e) Enriched score for signatures in pathways of cancer. (f) Enriched score for signatures in the pathway of VEGF signaling.

Through gene set enrichment analysis (GSEA), we tried to understand the differentially enriched signals between high and low immune-risk patients. The results showed that signatures, related to pathways of cell cycle, apoptosis, NOD like receptor signaling, Notch signaling, and VEGF signaling, were significantly enriched in high immune-risk patients, indicating the proliferative status of HCC cells (Figure 7, Table 2).

**Figure 8:**
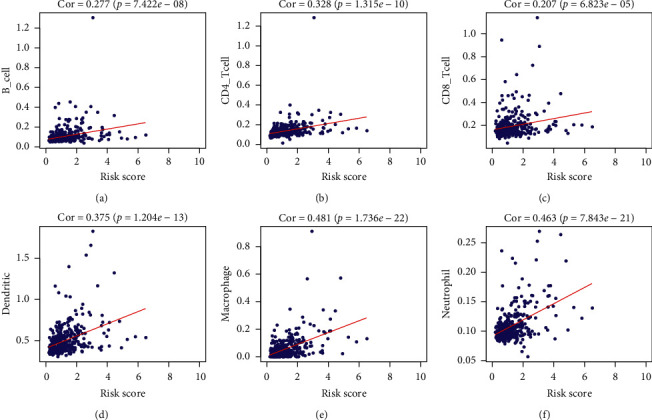
The correlation between immune-risk score and commonly infiltrated immune cells in HCC. (a) Relation between infiltrated B cell score and immune-risk score. (b) Relation between infiltrated CD4+ T cell score and immune-risk score. (c) Relations between infiltrated CD8+ T cell score and immune-risk score. (d) Relation between dendritic cell score and immune-risk score. (e) Relation between macrophage score and immune-risk score. (f) Relations between infiltrated neutrophil score and immune-risk score.

HCC infiltrated immune cell populations were also analyzed to find whether the infiltration patterns were related to immune-risk score. It turned out infiltration of six types of immune cells (B cell, CD4+ T cell, CD8+ T cell, dendritic cell, macrophage, and neutrophil) were positively related to immune-risk score (*p* < 0.05). High immune-risk score was correlated to high infiltration of immune cells, which could also be extrapolated as exhaustive immune status (Figure 8). Of the six cell types, macrophages and neutrophils showed the highest relevance and significance. Also, using ssGSEA analysis to evaluate immune status between groups, we found the overall immune activity score of different signals was relatively higher in high immune-risk patients. Expression levels of immune checkpoints were also higher in high immune-risk patients (Supplemental Figure 3).

**Figure 9:**
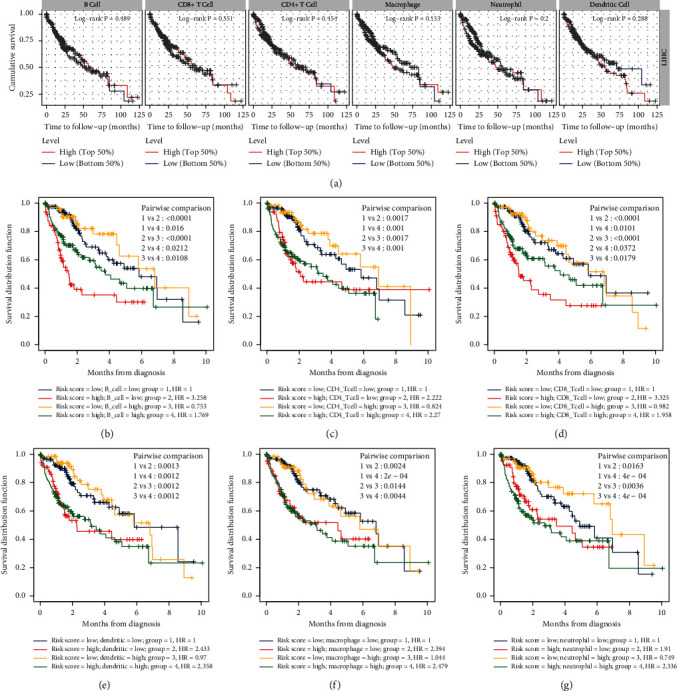
Prognostic value of immune-risk model was consistent between different immune-infiltration subgroups. (a) Survival difference was not obvious between subgroups of different immune infiltration groups of six immune cell types. (b) Survival difference between subgroups classified by B cell infiltration and immune-risk score levels. (c) Survival difference between subgroups classified by CD4 T cell infiltration and immune-risk score levels. (d) Survival difference between subgroups classified by CD8 T cell infiltration and immune-risk score levels. (e) Survival difference between subgroups classified by dendritic cell infiltration and immune-risk score levels. (f) Survival difference between subgroups classified by macrophage infiltration and immune-risk score levels. (g) Survival difference between subgroups classified by neutrophil infiltration and immune-risk score levels.

We further analyzed the survival difference between immune cell infiltration groups, and there was no significant difference between high and low infiltration groups of six immune cell types (Figure 9(a)). However, after assigning patients according to immune-score and each cell type infiltration score, we found high immune-risk score was related to worse prognosis consistently, which was independent on immune cell infiltration levels (Figures 9(b)–9(g)). The patients with high-risk score and low B cell, CD4 + T cell, CD8 + T cell, or dendritic cell showed the worst OS, especially high-risk score combined with low B cell or CD 8+ T cell (Figures 9(b)–9(e)). However, HCC patients with high-risk score and high macrophage presented the worst OS, while the patients with low-risk score and low macrophage had the best OS (Figure 9(f)). This is perhaps because that tumor-associated macrophage can promote cancer-related malignancy. HCC patients with high-risk score had worse OS than those with low-risk score, regardless of neutrophil level (Figure 9(g)).

### 3.2. Combined Nomogram Can Better Guide Treating Strategies in Clinical Practice

**Figure 10:**
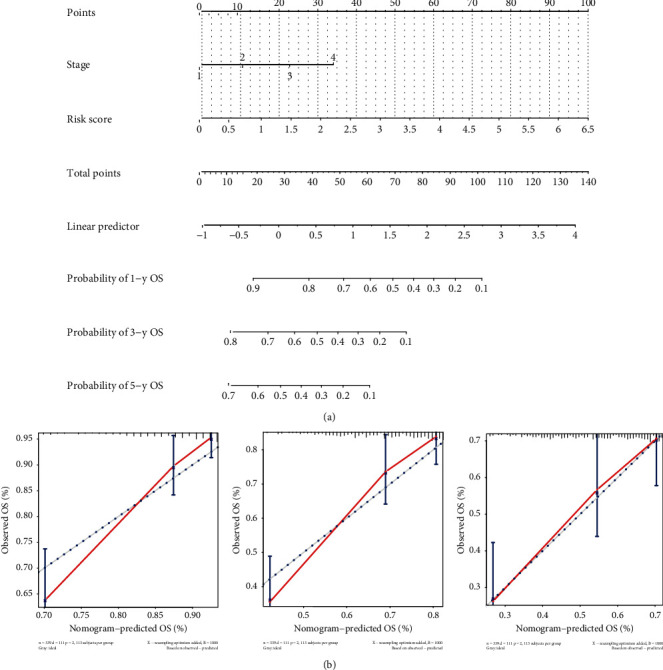
Construction of a nomogram with related factors for prognostic prediction. (a) Score model of nomogram in prediction of 1-, 3-, and 5-year overall survival. (b) Calibration graphs for 1-, 3-, and 5-year overall survival prediction within the nomogram (usage: the clinical stages and immune-risk score of patients were calculated and projected to the reference score bar, and total scores were compared to liner predictor to find the probability of 1-, 3-, and 5-year survival).

**Figure 11:**
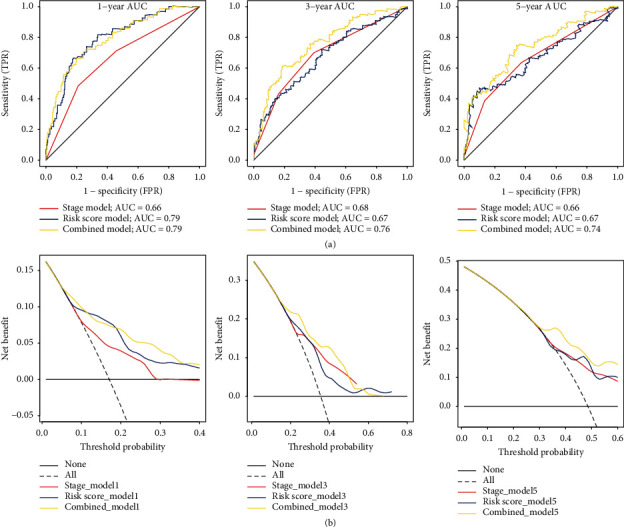
Comparison of prognostic values and clinical benefits between different models. (a) ROCs of prognostic models for 1-, 3-, and 5-year overall survival separately. (b) DCA curves of different models for 1-, 3-, and 5-year overall survival prediction. Dotted grey line represented all patients' benefits, and solid black line represented no benefits.

In consideration of all the survival-related factors, we generated a nomogram with tumor stages and immune-risk score to evaluate 1-, 3- and 5-year OS of HCC patients (Figure 10(a)). Calibration curves for the 3 models were also generated to show the consistence between estimation and actual probability (Figure 10(b)). The AUCs for the combined model in prediction of 1-, 3-, and 5-year OS were all over 0.7, which were better than the other two single-factor models (Figure 11(a)). DCAs of the three models also showed superiority of the combined model in prediction of 1-, 3-, and 5-year OS at various threshold probabilities (Figure 11(b)).

## 4. Discussion

In this study, we focused on the immune status of HCC to find the changed immune genes between HCC and relatively normal liver tissues, and through regression analysis, we further located eight survival-related signatures to construct an immune-risk score model for prognostic prediction. High score patients shared a worse outcome in comparison to the low-risk ones significantly, which was independent on immune infiltration levels, though high immune-risk score positively correlated with immune cell infiltration. The further constructed nomogram, using immune-risk score and tumor stages, could better predict the overall survival of patients in 1, 3, and 5 years than single factor, which is very useful for patient monitoring and instructive for choice of therapy in clinical practice. Though, many previous studies tried to find the differentially expressed prognostic biomarkers in HCC, immune-related models were seldom used to evaluate patients. However, with the development of immune therapy in cancer treatment, such as immune checkpoint blockade (ICB) and Car-T transfusion, immune-risk evaluation of cancer patients may better predict patients' survival and the following response to ICB or Car-T treatment.

In our study, MAPK signaling, Ras signaling, ErbB signaling, and EGFR tyrosine kinase inhibitor resistance pathways were enriched in high immune-risk patients, specifying the highly proliferative status of cancer cells in high immune-risk patients. Immune microenvironment of high immune-risk patients was more in favor of tumor growth. Also, though the immune-risk score was positively related to tumor infiltrating immune cells, high immune-risk patients tended to share a worse outcome after HCC resection, which may be due to exhaustive immune status. Former studies have shown that immune cell function depression was related to cancer progression, and we thought those high immune-risk patients could have better treating effect with ICB or Car-T therapy.

In the immune-risk model, secreted phosphoprotein 1 (SPP1 or OPN) is a widely studied signature in tumors, which is normally involved in the process of osteoclasts' attachment to mineralized bone matrix. It is elevated in tumors for progression and metastasis, and its alternatively spliced variants are related to many malignant traits in cancers, such as epithelial-mesenchymal plasticity, cancer cell stemness, chemoresistance, and radioresistance [15–20]. However, the variants of SPP1 due to aberrantly processing may cause autoimmune reactions in tumors, which can lead to better outcomes in partial patients [21, 22]. In HCC, SPP1 is a validated prognostic biomarker, and it may induce chemoresistance through regulation of autophagy in HCC cells [23, 24]. The next step of exploration shall focus on the involved regulatory pathways in order to find the potential drugs and inhibitors.

Two of the signatures in our immune-risk model were related to NF-kB and TGF-b signal pathways. Heat shock protein family A (Hsp70) member 4 (HSPA4) was formerly reported to involve inflammation responses and could be used as a biomarker for early lung cancer diagnosis and glioma outcome evaluation [25, 26]. It has been found extracellular HSP70 could activate ERK1/2, NF-kB, and proinflammatory genes transcription in lung cancer cell, and in breast cancer, tumor-educated B cells could target HSPA4 to promote lymph node metastasis through Src/NF-kB pathway [27, 28]. Also, Th2 cells in allergic disease could increase Hsp70, which was involved in pathogenesis [29]. N-myc downregulated 1 (NDRG1) was a tumor suppressor, which has been reported in various studies; it could attenuate NF-kB and TGF-b pathways in pancreatic cancer cells, and downregulated NDRG1 was related to increased proliferation, invasion, and migration of digestive cancers [30–33]. It additionally involves apoptosis, glycolytic, and lipid metabolism in cancer cells, and virus infection process was also related to NDRG1 [34–40]. Another signature, mitogen-activated protein kinase 2 (MAP4K2), was involved in MAPK signaling pathway, which corroborated with the enrichment results. We thought these pathways regulated by the three signatures involved crucial proliferation and progression process of tumors, and they all influence HCC tumor immune microenvironment through unrevealed mechanisms.

In the immune-risk model, bromodomain containing 8 (BRD8) was an androgen receptor coactivator, while TNF receptor associated factor 3 (TRAF3) was also involved in functioning of a variety of receptors. The knowledge of BRD8 in immune regulation was rare; however, it was related to p53-dependent apoptosis and could be a chemosensitizing target in colorectal cancer [41–43]. TRAF3 has been found to interplay with toll like receptors and TNF receptors in lymphocytes, and previous studies have found TRAF3 could attenuate noncanonical NF-kB pathway, influencing B cell and T cell development and recruitment through chemokine regulation [44–49]. Kit ligan (KITLG or stem cell factor, SCF) is a cytokine, which has been testified to be expressed by both cancer cells and immune cells and related to tumor growth, metastasis, and stemness [50, 51]. In HCC, KITLG has been found to be an independent prognostic factor, and it can bind to c-kit receptor expressed by various immune cell types, leading to pathological process of allergy [52–55]. High expressions of BRD8, TRAF3, and KITLG were related to hyperfunction of receptors in tumor or immune cell populations, which could in turn influence the survival of patients. We thought high expression of the three markers in HCC was related to exhaustive immune status, indicating highly communicative signals in tumor microenvironment, in which ICB treatment could yield more benefits.

Overall, the immune-risk model and the combined nomogram are of great value in clinical practice for prognostic prediction. The immune microenvironment difference between high and low immune-risk score patients may decide patients' responses to immune treatment, and understanding the immunogenetic changes or patterns of immune infiltration in HCC can optimize treating strategies and drug application [56].

There are several limitations about our investigation. Firstly, expression analysis through bioinformatic methods still needs tissue sample confirmation in consideration of other potential confounding factors, such as ethic bias. Also, the immune status behind immunogenetic changes requires further exploration of infiltrated immune cells, which were simply estimated in our study.

## 5. Conclusion

The immune-risk model, based on expression of SPP1, BRD8, NDRG1, KITLG, HSPA4, TRAF3, ITGAV, and MAP4K2, can efficiently differentiate HCC patients into high and low immune-risk subpopulations, and in combination with tumor stages, the derived nomogram can precisely predict the 1-, 3-, and 5-year overall survival among HCC patients, providing a tool for prognostic prediction.

## Figures and Tables

**Table 1:** Patients' information in the TCGA, ICGC, and GEO cohorts.

Clinical characteristics		Total (%)
TCGA		365
Survival status	Survival	239 (65.48)
	Death	126 (34.52)

Age	<61 years	173 (47.40)
	≧61 years	192 (52.60)

Gender	Male	246 (67.40)
	Female	119 (32.60)

Histological grade	G1	55 (15.07)
	G2	175 (47.95)
	G3	118 (32.33)
	G4	12 (3.29)
	Unknown	5 (1.36)

Stage	I	170 (46.58)
	II	84 (23.01)
	III	83 (22.74)
	IV	5 (1.10)
	Unknown	24 (6.57)

ICGC		232
Survival status	Survival	189 (81.47)
	Death	43 (18.53)

Age	<69 years	116 (50.00)
	≧69 years	116 (50.00)

Gender	Male	171 (73.71)
	Female	61 (26.29)

Stage	I	36 (15.52)
	II	106 (45.69)
	III	71 (30.60)
	IV	19 (8.19)

Prior malignancy	No	202 (87.07)
	Yes	30 (12.93)

GSE14520		209
Survival status	Survival	130 (62.20)
	Death	79 (37.80)

Age	<60 years	168 (80.38)
	≧60 years	41 (19.62)

Gender	Male	26 (12.44)
	Female	183 (87.56)

Stage (TNM)	I	90 (43.06)
	II	74 (35.41)
	III	43 (20.57)
	Unknown	2 (0.96)

Stage (CLIP)	0	95 (45.45)
	1	67 (32.57)
	2	32 (15.30)
	3	9 (4.30)
	4	3 (1.44)
	5	1 (0.48)
	Unknown	2 (0.96)

**Table 2:** Gene sets enriched in phenotype high.

NAME	ES	NES	FDR q-val
KEGG_PYRIMIDINE_METABOLISM	0.672451	2.173925	0
KEGG_CELL_CYCLE	0.737056	2.097065	7.27E-04
KEGG_P53_SIGNALING_PATHWAY	0.620247	2.06442	0.001371
KEGG_BASAL_TRANSCRIPTION_FACTORS	0.709419	2.012458	0.003543
KEGG_PATHWAYS_IN_CANCER	0.551037	1.914016	0.007377
KEGG_NOTCH_SIGNALING_PATHWAY	0.663903	1.91001	0.007401

## Data Availability

The datasets generated and/or analyzed during the current study are available in the TCGA (https://portal.gdc.cancer.gov/), GEO (https://www.ncbi.nlm.nih.gov/geo/), and ICGC (https://icgc.org/) repository.

## Additional Points

*Summary*. Hepatocellular carcinoma has been one of the most malignant tumors, conflicting a large number of patients worldwide with poor responses to drugs. The high recurrent rate after resection makes outcomes of patients even worse. Though new immune targeting drugs approved by federal government showed some light in treatment, the overall responses among patients are still unsatisfactory. As a result, the prognostic prediction of patients is very important in consideration of treating strategies and patient monitoring. Former studies tried to find the biomarkers in HCC for diagnosis and prognosis, the scope of immunogenetic changes have seldom been investigated. Our study managed to find the significantly changed immune signatures in HCC, and based on survival-related immune genes, we constructed an immune-risk model for prognostic prediction. The high-risk patients shared worse outcomes, and the enriched pathways demonstrated the protumor growth immune microenvironment. Also, the prognostic value of immune-risk score in HCC patients was independent on immune infiltration levels, while it did positively correlate with infiltrating immune cells, indicating high-risk patients may have better ICB therapy efficacy with immune exhaustive status. In combination with tumor stages, the nomogram showed high accuracy in prediction of 1-, 3-, and 5- year overall survival, which is valuable for clinical evaluation in HCC treatment.

## Supplementary Materials

Supplementary Materials Supplemental Figure 1. Differentially expressed transcription factors in HCC tissues and the regulation network between transcription factors and immune signatures. Supplemental Figure 2. PFS difference between high and low immune-risk score patients in TCGA dataset and the respective prognostic values for 0.5-, 1-, 2-, 3-, and 5-year survival. Supplemental Figure 3. Comprehensive immune status evaluation of high and low immune-risk HCC patients.

## References

[1] Torre L. A., Bray F., Siegel R. L., Ferlay J., Lortet-Tieulent J., Jemal A. (2012). Global cancer statistics. *CA: A Cancer Journal for Clinicians*.

[2] Hartke J., Johnson M., Ghabril M. (2017). The diagnosis and treatment of hepatocellular carcinoma. *Seminars in Diagnostic Pathology*.

[3] Jindal A., Thadi A., Shailubhai K. (2019). Hepatocellular carcinoma: etiology and current and future drugs. *Journal of Clinical and Experimental Hepatology*.

[4] Chen L., Han X. (2015). Anti-PD-1/PD-L1 therapy of human cancer: past, present, and future. *Journal of Clinical Investigation*.

[5] Long J., Zhang L., Wan X. (2018). A four-gene-based prognostic model predicts overall survival in patients with hepatocellular carcinoma. *Journal of Cellular and Molecular Medicine*.

[6] Wan Z., Zhang X., Luo Y., Zhao B. (2019). Identification of hepatocellular carcinoma-related potential genes and pathways through bioinformatic-based analyses. *Genetic Testing and Molecular Biomarkers*.

[7] Hudson T. J., Hudson T. J., Anderson W (2010). International network of cancer genome projects. *Nature*.

[8] Tomczak K., Czerwinska P., Wiznerowicz M. (2015). The Cancer Genome Atlas (TCGA): an immeasurable source of knowledge. *Contemporary Oncology (Poznan, Poland)*.

[9] Ritchie M. E., Phipson B., Wu D. (2015). Limma powers differential expression analyses for RNA-sequencing and microarray studies. *Nucleic Acids Research*.

[10] Yu G., Wang L.-G., Han Y., He Q.-Y. (2012). clusterProfiler: an R package for comparing biological themes among gene clusters. *OMICS: A Journal of Integrative Biology*.

[11] Mootha V. K., Lindgren C. M., Eriksson K.-F. (2003). PGC-1*α*-responsive genes involved in oxidative phosphorylation are coordinately downregulated in human diabetes. *Nature Genetics*.

[12] Subramanian A., Tamayo P., Mootha V. K. (2005). Gene set enrichment analysis: a knowledge-based approach for interpreting genome-wide expression profiles. *Proceedings of the National Academy of Sciences*.

[13] Li B., Severson E., Pignon J. C. (2016). Comprehensive analyses of tumor immunity: implications for cancer immunotherapy. *Genome Biology*.

[14] Li T., Fan J., Wang B. (2017). TIMER: a web server for comprehensive analysis of tumor-infiltrating immune cells. *Cancer Research*.

[15] Jia R., Liang Y., Chen R. (2016). Osteopontin facilitates tumor metastasis by regulating epithelial-mesenchymal plasticity. *Cell Death & Disease*.

[16] Hao C., Cui Y., Hu M. (2017). OPN-a splicing variant expression in non-small cell lung cancer and its effects on the bone metastatic abilities of lung cancer cells in vitro. *Anticancer Research*.

[17] Xu C., Sun L., Jiang C. (2017). SPP1, analyzed by bioinformatics methods, promotes the metastasis in colorectal cancer by activating EMT pathway. *Biomedicine & Pharmacotherapy*.

[18] D’Addazio G., Artese L., Traini T., Rubini C., Caputi S., Sinjari B. (2018). Immunohistochemical study of osteopontin in oral squamous cell carcinoma allied to fractal dimension. *Journal of Biological Regulators and Homeostatic Agents*.

[19] Assidi M., Gomaa W., Jafri M. (2019). Prognostic value of Osteopontin (SPP1) in colorectal carcinoma requires a personalized molecular approach. *Tumour Biology:The Journal of the International Society for Oncodevelopmental Biology and Medicine*.

[20] Gimba E. R. P., Brum M. C. M., Nestal De Moraes G. (2019). Full-length osteopontin and its splice variants as modulators of chemoresistance and radioresistance (Review). *International Journal of Oncology*.

[21] Kamal A., Darwish R. K., Saad S. (2017). Association of osteopontin gene polymorphisms with colorectal cancer. *Cancer Investigation*.

[22] Alsarkhi L. K., Weber G. F. (2018). Antiosteopontin autoantibodies in various types of cancer. *Oncology Reports*.

[23] Liu G., Fan X., Tang M. (2016). Osteopontin induces autophagy to promote chemo-resistance in human hepatocellular carcinoma cells. *Cancer Letters*.

[24] Cabiati M., Gaggini M., Cesare M. M. (2017). Osteopontin in hepatocellular carcinoma: a possible biomarker for diagnosis and follow-up. *Cytokine*.

[25] Tang T., Yang C., Brown H. E., Huang J. (2018). Circulating heat shock protein 70 is a novel biomarker for early diagnosis of lung cancer. *Disease Markers*.

[26] Lämmer F., Delbridge C., Würstle S. (2019). Cytosolic Hsp70 as a biomarker to predict clinical outcome in patients with glioblastoma. *PLoS One*.

[27] Somensi N., Brum P. O., de Miranda Ramos V. (2017). Extracellular HSP70 activates ERK1/2, NF-kB and pro-inflammatory gene transcription through binding with RAGE in A549 human lung cancer cells. *Cellular Physiology and Biochemistry*.

[28] Gu Y., Liu Y., Fu L. (2019). Tumor-educated B cells selectively promote breast cancer lymph node metastasis by HSPA4-targeting IgG. *Nature Medicine*.

[29] Min H. J., Kim K. S., Yoon J.-H., Kim C.-H., Cho H.-J. (2017). T-helper 2 cytokine-induced heat shock protein 70 secretion and its potential association with allergic rhinitis. *International Forum of Allergy & Rhinology*.

[30] Menezes S. V., Fouani L., Huang M. L. H. (2019). The metastasis suppressor, NDRG1, attenuates oncogenic TGF-*β* and NF-*κ*B signaling to enhance membrane E-cadherin expression in pancreatic cancer cells. *Carcinogenesis*.

[31] Cen G., Zhang K., Cao J., Qiu Z. (2017). Downregulation of the N-myc downstream regulated gene 1 is related to enhanced proliferation, invasion and migration of pancreatic cancer. *Oncology Reports*.

[32] Chen K., Liu X.-H., Wang F.-R., Liu H.-P., Huang Z.-P., Chen X. (2018). The prognostic value of decreased NDRG1 expression in patients with digestive system cancers. *Medicine*.

[33] Dai T., Dai Y., Murata Y. (2018). The prognostic significance of N-myc downregulated gene 1 in lung adenocarcinoma. *Pathology International*.

[34] Guo D.-D., Xie K.-F., Luo X.-J. (2020). Hypoxia-induced elevated NDRG1 mediates apoptosis through reprograming mitochondrial fission in HCC. *Gene*.

[35] Liu W., Zhang B., Hu Q. (2017). A new facet of NDRG1 in pancreatic ductal adenocarcinoma: suppression of glycolytic metabolism. *International Journal of Oncology*.

[36] Sevinsky C. J., Khan F., Kokabee L., Darehshouri A., Maddipati K. R., Conklin D. S. (2018). NDRG1 regulates neutral lipid metabolism in breast cancer cells. *Breast Cancer Research*.

[37] Yang X., Zhu F., Yu C. (2017). N-myc downstream-regulated gene 1 promotes oxaliplatin-triggered apoptosis in colorectal cancer cells via enhancing the ubiquitination of Bcl-2. *Oncotarget*.

[38] Chen L., Xing C., Ma G. (2018). N-myc downstream-regulated gene 1 facilitates influenza A virus replication by suppressing canonical NF-*κ*B signaling. *Virus Research*.

[39] Gupta P., Shahzad N., Harold A. (2020). Merkel cell polyomavirus downregulates N-myc downstream-regulated gene 1, leading to cellular proliferation and migration. *Journal of Virology*.

[40] Schweitzer C. J., Zhang F., Boyer A., Valdez K., Cam M., Liang T. J. (2017). N-myc downstream-regulated gene 1 restricts hepatitis C virus propagation by regulating lipid droplet biogenesis and viral assembly. *Journal of Virology*.

[41] Jiang C.-Y., Ruan Y., Wang X.-H. (2016). MiR-185 attenuates androgen receptor function in prostate cancer indirectly by targeting bromodomain containing 8 isoform 2, an androgen receptor co-activator. *Molecular and Cellular Endocrinology*.

[42] Lashgari A., Fauteux M., Maréchal A., Gaudreau L. (2018). Cellular depletion of BRD8 causes p53-dependent apoptosis and induces a DNA damage response in non-stressed cells. *Scientific Reports*.

[43] Yamada H. Y., Rao C. V. (2009). BRD8 is a potential chemosensitizing target for spindle poisons in colorectal cancer therapy. *International Journal of Oncology*.

[44] Bishop G. A., Stunz L. L., Hostager B. S. (2018). TRAF3 as a multifaceted regulator of B lymphocyte survival and activation. *Frontiers in immunology*.

[45] Häcker H., Tseng P.-H., Karin M. (2011). Expanding TRAF function: TRAF3 as a tri-faced immune regulator. *Nature Reviews Immunology*.

[46] Yi Z., Wallis A. M., Bishop G. A. (2015). Roles of TRAF3 in T cells: many surprises. *Cell Cycle*.

[47] Hildebrand J. M., Yi Z., Buchta C. M., Poovassery J., Stunz L. L., Bishop G. A. (2011). Roles of tumor necrosis factor receptor associated factor 3 (TRAF3) and TRAF5 in immune cell functions. *Immunological Reviews*.

[48] Liu F., Cheng L., Xu J., Guo F., Chen W. (2018). miR-17-92 functions as an oncogene and modulates NF-*κ*B signaling by targeting TRAF3 in MGC-803 human gastric cancer cells. *International Journal of Oncology*.

[49] Perez-Chacon G., Adrados M., Vallejo-Cremades M. T., Lefebvre S., Reed J. C., Zapata J. M. (2018). Dysregulated TRAF3 and BCL2 expression promotes multiple classes of mature non-hodgkin B cell lymphoma in mice. *Frontiers in immunology*.

[50] Shen S. Q., Wang R., Huang S. G. (2017). Expression of the stem cell factor in fibroblasts, endothelial cells, and macrophages in periapical tissues in human chronic periapical diseases. *Genetics and Molecular Research*.

[51] Wang L., Wang J., Li Z. (2016). Silencing stem cell factor attenuates stemness and inhibits migration of cancer stem cells derived from Lewis lung carcinoma cells. *Tumor Biology*.

[52] Wang X., Ren H., Zhao T. (2014). Stem cell factor is a novel independent prognostic biomarker for hepatocellular carcinoma after curative resection. *Carcinogenesis*.

[53] Fonseca W., Rasky A. J., Ptaschinski C. (2019). Group 2 innate lymphoid cells (ILC2) are regulated by stem cell factor during chronic asthmatic disease. *Mucosal Immunology*.

[54] Cho K.-A., Park M., Kim Y.-H., Woo S.-Y. (2017). Th17 cell-mediated immune responses promote mast cell proliferation by triggering stem cell factor in keratinocytes. *Biochemical and Biophysical Research Communications*.

[55] Al-Azzam N., Kondeti V., Duah E., Gombedza F., Thodeti C. K., Paruchuri S. (2015). Modulation of mast cell proliferative and inflammatory responses by leukotriene D4and stem cell factor signaling interactions. *Journal of Cellular Physiology*.

[56] Chen Q.-F., Li W., Wu P.-H., Shen L.-J., Huang Z.-L. (2019). Significance of tumor-infiltrating immunocytes for predicting prognosis of hepatitis B virus-related hepatocellular carcinoma. *World Journal of Gastroenterology*.

